# Structure Predictions of Two *Bauhinia variegata* Lectins Reveal Patterns of C-Terminal Properties in Single Chain Legume Lectins

**DOI:** 10.1371/journal.pone.0081338

**Published:** 2013-11-19

**Authors:** Gustavo M. S. G. Moreira, Fabricio R. Conceição, Alan J. A. McBride, Luciano da S. Pinto

**Affiliations:** Centro de Desenvolvimento Tecnológico, Núcleo de Biotecnologia, Universidade Federal de Pelotas, Pelotas, Rio Grande do Sul, Brazil; Russian Academy of Sciences, Institute for Biological Instrumentation, Russian Federation

## Abstract

*Bauhinia variegata* lectins (BVL-I and BVL-II) are single chain lectins isolated from the plant *Bauhinia variegata*. Single chain lectins undergo post-translational processing on its N-terminal and C-terminal regions, which determines their physiological targeting, carbohydrate binding activity and pattern of quaternary association. These two lectins are isoforms, BVL-I being highly glycosylated, and thus far, it has not been possible to determine their structures. The present study used prediction and validation algorithms to elucidate the likely structures of BVL-I and -II. The program Bhageerath-H was chosen from among three different structure prediction programs due to its better overall reliability. In order to predict the C-terminal region cleavage sites, other lectins known to have this modification were analysed and three rules were created: (1) the first amino acid of the excised peptide is small or hydrophobic; (2) the cleavage occurs after an acid, polar, or hydrophobic residue, but not after a basic one; and (3) the cleavage spot is located 5-8 residues after a conserved Leu amino acid. These rules predicted that BVL-I and –II would have fifteen C-terminal residues cleaved, and this was confirmed experimentally by Edman degradation sequencing of BVL-I. Furthermore, the C-terminal analyses predicted that only BVL-II underwent α-helical folding in this region, similar to that seen in SBA and DBL. Conversely, BVL-I and -II contained four conserved regions of a GS-I association, providing evidence of a previously undescribed X4+unusual oligomerisation between the truncated BVL-I and the intact BVL-II. This is the first report on the structural analysis of lectins from *Bauhinia* spp. and therefore is important for the characterisation C-terminal cleavage and patterns of quaternary association of single chain lectins.

## Introduction

Lectins, also known as agglutinins, are proteins or glycoproteins capable of binding mono- or oligosaccharides in a specific, reversible, manner [1]. Although these proteins are present in various species [2], more information is available for plant lectins as they are usually easier to isolate and characterise [3]. Numerous experiments have demonstrated their diverse applications, including e.g.: insecticidal, antifungal, antiviral, antitumor, and immunomodulatory activities [3–5]. As their recognition of sugars is highly specific, lectins are used in glycobiology to study protein-carbohydrate interactions. These *in vitro* studies usually include the molecular characterisation of the lectin, which determines the three dimensional (3D) structure and thus the basis of its activity [6].

 Lectins are primarily synthesized as inactive precursors and are activated by two distinct processes. Legume lectins such as Concanavalin A (ConA) and *Canavalia brasiliensis* lectin (ConBr) undergo a complex post-translational modification process of deglycosylation, endoproteolytic cleavage, and polypeptide chain rebind. In this process, after excision of the N-terminal signal sequence, a linker and a C-terminal extension peptides, the β and γ chains, are linked by a peptide bond to form an inverted active lectin called the α chain [7-9]. Other lectins from the *Canavalia* [10], *Dioclea* [11–15] and *Cratylia* [16,17] genera are processed in a similar manner. However, other kind of lectins shows a simpler process, which is based on removal of the N-terminal signal peptide followed by cleavage of the C-terminal peptide [18-20]. This type of post-translational modification does not involve rebinding polypeptide chains and results in the production of single chain lectins such as the soybean agglutinin (SBA) [21], *Dolichos biflorus* lectin (DBL) [22,23], peanut agglutinin (PNA) [20], *Erythrina corallodendron* lectin (EcorL) and others from *Erythrina* spp. [24]. In addition to the post-translational modification, lectins can differ on their type of quaternary association, which can be defined as Canonical, ECorL-type, GS4-type, DBL-type, ConA- type, PNA-type, GS1-type, DB58-type, or Arcelin-5-type. Different interfaces for dimers (II, X1, X3, and X4) and tetamers (II+X1, II+X2, X4+unusual, and II+X4+unusual) are defined for each of these associations [18,25].

 The *Bauhinia variegata* lectins, BVL-I and BVL-II, are Gal/GalNAc specific single chain proteins [26] that have the capacity to promote skin regeneration [27], and to inhibit the adhesion of oral bacteria, thereby impairing biofilm formation [28]. Although several approaches exist for crystallisation [29], their three dimensional (3D) structures have not been resolved experimentally as they are difficult to crystalize, possibly due to the presence of different oligomerisation states of BVL-I and -II isoforms after purification. This way, their tertiary and quaternary structures could not be completely explored. As an alternative, *in silico* methodologies can be used to generate 3D predictive models of protein structures [30]. One such method applies homology-based algorithms in which 3D models are calculated using an existing, highly identical, structure from the Protein Data Bank (PDB) [31]. Using this approach, reliable 3D models can be calculated when the sequence identity is >30%, although >50% is recommended [32]. However, only approximately 0.7% of the available protein sequences have been structurally resolved experimentally [30]. Thus, when there are no or only low-identity templates available, *de novo* or *ab initio* protein modelling can be employed [33]. Regardless of the method used to predict a 3D structure, it is necessary to verify its accuracy through analysing amino acid interactions, stereochemistry, and structural similarity to the template [34]. A Ramachandran plot (RP), for example, shows which values of the Phi and Psi angles are possible for each amino acid residue in a protein, thereby indicating the percentage of amino acids in acceptable positions in the 3D model [35]. Another measurement of accuracy is the root-mean-square deviation (RMSD), which calculates the distance between the atoms of two superimposed protein structures [36]. Low RMSD values indicate that a given prediction is more reliable. When a template with high (>50%) or medium (30-50%) identity is used, the expected RMSD value for high-quality models is 1 and 2.5 Å, respectively [30,34].

In this study, lectins that undergo C-terminal processing (SBA, DBL, PNA and EcorL) were used to predict potential cleavage sites in BVL-I and -II. The structure prediction program Bhageerath-H was evaluated and chosen to generate structures for all the analysed lectins. By comparing the BVL-I and -II sequences and their predicted tertiary structures with the other lectins, it was possible to predict their quaternary structures. Additionally, the predicted BVL-I processing site was confirmed by Edman degradation sequencing. This is the first report describing a structural basis for lectins from *Bauhinia* spp. and the first description of the use of structure prediction and validation programs to study post-translational cleavage in lectins.

## Materials and Methods

### Amino acid sequences and protein structures

The amino acid sequences and structures of the analysed lectins were downloaded from GenBank and the Protein Data Bank (PDB), respectively, using the accession numbers listed in Table S1.

### Sequence analyses

The levels of similarity and identity between the analysed proteins were determined using EMBOSS Needle at the default settings, which is based on the BLOSUM62 matrix [37]. The multiple sequence alignment was calculated using Clustal Omega [38] at the default parameters. The quaternary association definitions were based on the previously described conserved sequences [18].

### Structure prediction and reliability

The amino acid sequence of each lectin (SBA, DBL, PNA, EcorL, BVL-I and -II) were used as the query sequence in three structure prediction programs: SwissModel [39], 3D Jigsaw [40] and Bhageerath-H [41]. The default settings were used without predetermined templates. The N-terminal signal peptides of the amino acid sequences were not included in the analysis. For the additional structural analyses of the C-terminal regions of BVL-I and -II, the last 15 amino acid residues were excluded from the original protein sequences. 

The quality of the structures were analysed using QMEAN [42] and PROCHECK [35]. QMEAN generated the Z- and QMEAN scores and PROCHECK produced the RP. Swiss-PDB Viewer v4.0.4 software [43] was used to calculate the RMSD of the predicted structures compared to their respective PDB templates by selecting <Calculate RMS> for the Cα backbone option after <Magic Fit>. Thus, four quality measurements (see Table S3) were considered in determining the best prediction of the five structures generated by Bhageerath-H.

### Program selection and definition of C-terminal cleavage sites

The best prediction program was identified by analysing the average Z- and QMEAN scores, the RP and the RMSD (see Table S4) as well as the number of amino acids excluded during prediction and the coherence of the legume lectins β-sheets prediction for the modelled structures (see Figures S1 and S2). Only the best Bhageerath-H predictions, selected as described in the last section, were considered. For further analysis of the cleavage sites, both the position, based on the multiple sequence alignment, and the properties of each amino acid [44] were evaluated in order to identify patterns in the C-terminal region of BVL-I and -II.

### Structural error evaluation

After running the QMEAN of the predicted and PDB structures, the local error of each structure was determined. A black horizontal line was used to indicate the maximum local error in each of the PDB structures, and values below this line were considered insignificant in the predicted structures and therefore likely to be present in the native lectin. Conversely, error values above the line indicated an unreliable region that was unlikely to be present in the native protein structure.

### Visualization and imaging of structures

Manipulation and acquisition of the images from the predicted structures was carried out using PyMol v1.3r1 [45].

### Determination of the BVL-I sequence

The *B. variegata* lectins were obtained as previously described [26]. Briefly, the plant seeds were ground and suspended in a Tris-HCl 50 mM, sodium chloride 150 mM buffer (Tris-HCl + NaCl pH 7.6). After 24 h agitation, the mixture was centrifuged (20 min; 3500 × *g*), the supernatant was recovered and precipitated with 0-60% ammonium sulphate. After 3h at 4°C, the precipitate was collected by centrifugation and the pellet was suspended and dialyzed against Tris-HCl + NaCl pH 7.6. Purification by affinity chromatography was carried out using an agarose-lactose matrix (Sigma Aldrich) and a glycine 50 mM, sodium chloride 150 mM (pH 2.6) elution buffer. The fractions containing the protein were dialyzed in water, lyophilized and stored at -20°C until use. Only the BVL-I sequence was detected by Edman degradation performed with an Applied Biosystems 477A sequencer according to the manufacturer’s instructions.

## Results

### Selection of the 3D prediction program

 The SwissModel (SM) 3D structures presented acceptable quality, and the carbon backbone was identical to the used templates from PDB. However, some amino acids from the query sequences were automatically excluded by the program, and thus preventing a complete analysis of the lectins. Similarly, although 3D Jigsaw (3DJ) predicted the correct β-sheets for SBA, it was not able to predict the correct 3D structures for BVL-II, DBL, PNA and EcorL due to the high number of excluded amino acids. Of note, the Bhageerath-H (BH) program did not exclude any amino acids from any of the analysed sequences. Of the five structures generated by this program for each protein, the most reliable was selected based on the analysis of four parameters: Z-score, QMEAN score, RP and RMSD (see Table S3). All the 3D structures predicted by BH included the characteristic legume lectins β-sheets (Figures S1 and S2). Therefore, based on the overall reliability of the predicted 3D structures summarized in Table 1 (see Table S4 for additional information), the BH program was chosen to perform further studies on BVL-I and -II and on the other single chain lectins.

**Table 1:** Overall reliability of the evaluated prediction programs.

Prediction program	Number of excluded amino acids	Z-score**^*a*^**	QMEAN score**^*b*^**	% RP**^*c*^**	RMSD (Å)**^*d*^**	β-sheets prediction**^*f*^**
SwissModel	18-25	Good	0.78	85.8	0.26**^*e*^**	Coherent
3D Jigsaw	2-142	Medium	0.64	69.8	1.92	Incoherent
Bhageerath-H	None	Good	0.71	88.9	0.79	Coherent

*a* Expected Z-score is: |Z-score|<1 for good predictions, 1<|Z-score|<2 for medium predictions, and |Z-score|>2 for bad predictions;

*b* Expected QMEAN score is ≈1;

*c* Expected RP value is >90%, but the values from PDB structures were used as reference;

*d* Expected RMSD value is <2.5Å;

*e* Expected RMSD value is <1Å;

*f* Based on the structures shown in Figures S1 and S2.

### Identification of the C-terminal cleavage sites

**Figure 1:**
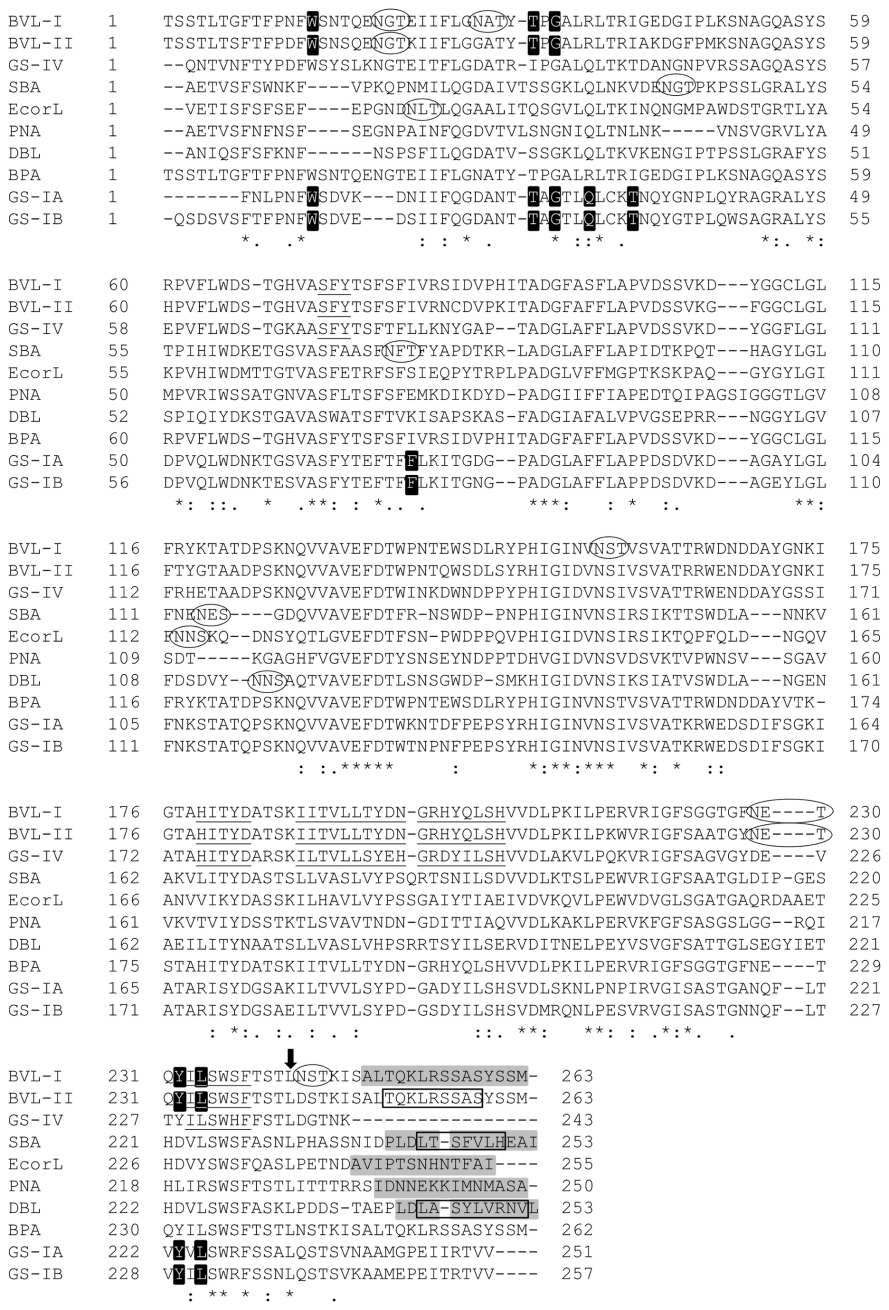
Protein sequence alignment of the studied lectins. All lectins with defined C-terminal processing have their cleavage spot located 5-8 amino acids after the conserved Leu indicated by the arrow. This spot is just after an acid, polar or hydrophobic amino acid (Ser, Asn and Pro). Moreover, the cleaved peptide starts and ends with hydrophobic/small amino acids (Pro, Leu, Ala, Ile and Met). Ellipses (○), glycosylation sites. Grey highlights, amino acids in the BVL-I sequence which were not detected by Edman sequencing (starts between Ser248 and Ala249), and amino acids cleaved from the C-terminal region of the other lectins. Underlined, conserved pattern of GS-IV quaternary association. Black highlights, conserved pattern of GS-I quaternary association. Open box (□), amino acids from the C-terminal α-helix of BVL-II/BHα, SBA/BH and DBL (PDB entry: 1BJQ, chain A). Asterisk (*), conserved amino acids. Colon (:), amino acids with strong similar properties. Period (.), amino acids with weak similar properties.

By analysing the multiple sequence alignment of the lectins that are cleaved at their C-terminus (SBA, EcorL, PNA and DBL, see Figure 1), it was possible to identify conserved characteristics. First, the cleaved C-terminal peptide always started and ended with either a hydrophobic or a small amino acid (Pro, Ala, Ile or Leu). Second, the cleavage site was located 5-8 amino acids after a Leu residue that was conserved in all lectins. Finally, this cleavage occurs immediately after an acidic (Asn in SBA and EcorL), polar (Ser in PNA) or hydrophobic (Pro in DBL) amino acid, and never after a basic residue such as Arg or Lys (Figure 1, see also Text S1). Based on these criteria, BVL-I and -II were predicted to contain a C-terminal cleavage site between Ser248 and Ala249. 

### Assessment of structural error characteristics

**Figure 2:**
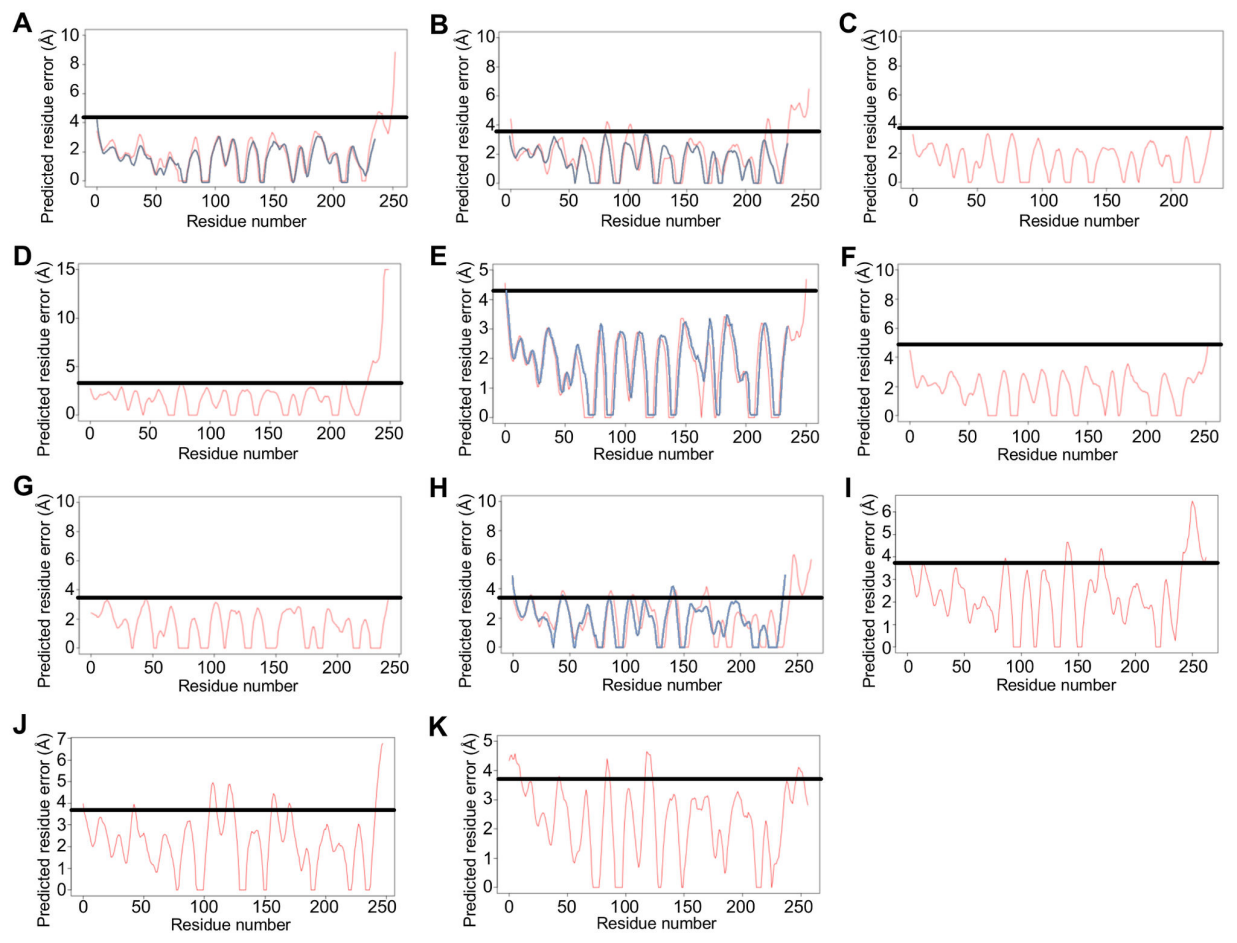
Local errors for the PDB structures and the predictions with and without the C-terminal peptide. All lectins were submitted to QMEAN analyses to determine their reliability. The horizontal line represents the maximum acceptable local error for the predictions based on their respective PDB structure value. The elevated local error characterizes a pattern of cleavage in SBA, EcorL and PNA, while DBL shows a pattern of stable C-terminal region by the presence of an α-helix. This information served to identify the structural behaviour of the C-terminal region from BVL-I, which is a truncated form, and BVL-II, which is an intact form. (A) SBA (PDB entry: 1SBF, blue curve) and SBA/BH (red curve). (B) EcorL (PDB entry: 1AX0, blue curve) and EcorL/BH (red curve). (C) PNA (PDB entry: 1CIW). (D) PNA/BH. (E) DBL:A (PDB entry: 1BJQ, chain A) in red and DBL:C (PDB entry 1BJQ, chain C) in blue. (F) DBL/BH. (G) GS-IV (PDB entry: 1LEC). (H) BVL-I/BH1 (red curve) and BVL-I/BH2 (blue curve). (I) BVL-II/BH1, which had an exacerbated C-terminal local error similar to BVL-I/BH1. (J) BVL-II/BH2, in which the exclusion of 15 last amino acids did not interfere on C-terminal local error. (K) BVL-II/BHα, in which the presence of a C-terminal α-helix reduced the local error on this region of the structure.

**Figure 3:**
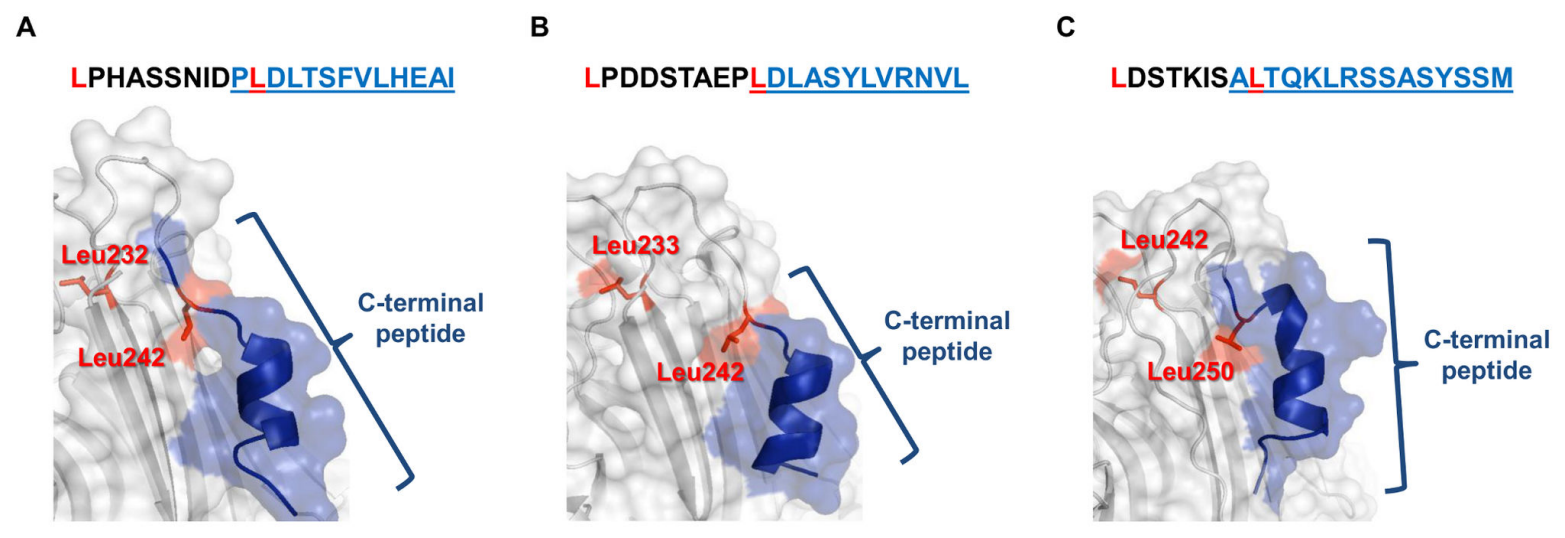
Constitution of the C-terminal peptide and its structural representation in SBA, DBL and BVL-II. The underlined C-terminal peptide of SBA (A), DBL (B) and BVL-II (C) has hydrophobic/small amino acids at its start (Pro, Leu and Ala, respectively) and end (Ile, Leu and Met, respectively). Moreover, these three lectins are cleaved in the region afterward 5-8 amino acids of the conserved Leu (red, in positions 232, 233 and 242, respectively) right after Asn240 (SBA), Pro241 (DBL) or Ser248 (BVL-II). The cleaved C-terminal peptide shown underlined has its first Leu (red, positions 242, 242 and 250, respectively) with buried side chain in all three lectins. Since the analysed EcorL and PNA do not show any Leu on their C-terminal peptide, this amino acid may play an important role to stabilize the C-terminal α-helix and thus avoiding its cleavage.

The C-terminal regions contained exacerbated local error peaks in the 3D models of SBA (SBA/BH), EcorL (EcorL/BH) and PNA (PNA/BH) that were not present in the corresponding PBD structures (Figures 2a-d). Of note, there was no evidence of local errors in the DBL prediction (DBL/BH, Figure 2e, f). Another important observation was that the EcorL and PNA C-terminal regions contains two and three Asn residues, respectively, while DBL had one Asn and SBA has none (see Figure 1). Furthermore, only SBA and DBL contain Leu residues (3 and 4, respectively) and a low number of Ile (one and zero, respectively). Also, the first Leu of the C-terminal peptide had a buried side chain and a conserved position in both 3D structures (Figure 3a, b). These characteristics divided the analysed lectins into two distinct groups: one including EcorL and PNA; and other DBL and SBA (Table S5).

Similar to SBA, EcorL and PNA, the C-terminal region of the predicted BVL-I/BH1 and BVL-II/BH1 did not exhibit globular folding. Furthermore, the local errors were insignificant across most of the amino acids, except for this region when GS-IV values were used as reference (Figures 2g-i). To improve the C-terminal analyses, additional predictions were made without the predicted C-terminal peptide region, which corresponded to 15 amino acids (BVL-I/BH2 and BVL-II/BH2). For BVL-I, the absence of the C-terminal peptide reduced the local error in this region (Figure 2h), while for BVL-II the local error increased slightly (Figure 2i, j). Interestingly, an alternative structure for BVL-II (BVL-II/BHα), which was initially discarded, included a C-terminal α-helix that was not predicted in the BVL-I, PNA or EcorL 3D models (including the discarded ones). An error in one strand of the front sheet explained the low quality measurements observed in BVL-II/BHα (Figure S1, Table S4). Nonetheless, the local error in the C-terminal region was significantly reduced and thus the scores stayed near the maximum (Figure 2k).

These data showed that the C-terminal region of BVL-I behaved the same way as those from EcorL, PNA and SBA. The high local error profile in the C-terminal region, which was reduced after the exclusion of this region, was present in these three lectins. Although BVL-II has high sequence identity to BVL-I (87.8%), the predicted behaviour of the C-terminus was different, once the high local errors were not reduced by exclusion of this region. Rather, the presence of an α-helix in this region reduced most of the elevated local error. This situation was similar to the pattern of the non-truncated (DBL:A) and truncated (DBL:C) variants of DBL in the PDB, in which DBL:A had no relevant local errors when the maximum value from DBL:C was used as the reference (Figure 2e). In addition, in both BVL-I and -II, the C-terminal region demonstrated the same amino acid profiles of SBA and DBL, as there were two Leu residues and no Asn or Ile (Table S5). This could have permitted the formation of a stable α-helix and may have been the reason for the high structural homology between BVL-II, SBA/BH and DBL:A (Figure 3).

### BVL-I sequence analysis

Edman sequencing was performed in order to provide experimental evidence for some of the *in silico* findings. Only BVL-I was detected and the resulting sequence indeed did not contain the 15-amino acid C-terminal region (see Figure 1). This data confirmed the *in silico* analyses that predicted the presence of a cleavage site in the C-terminal region of this lectin.

## Discussion

The majority of the lectins with previously defined 3D structures included in this study contain up to three glycosylation sites and rarely include divergent isoforms (Figure 1). However, one of the BVL lectins isoforms (BVL-I) is highly glycosylated (five sites) and the probable arbitrary formation of dimers and tetramers with BVL-II may be the reason for the difficulties on crystallography experiments. To overcome this type of problem, various methods are available to study and identify patterns in these proteins *in silico*. These methodologies predict structural information based on the protein sequence, generating the secondary, tertiary or quaternary structures of a target protein. These approaches use individual programs, which differ in their algorithms and reliability [46]. This has made it possible to predict protein aggregation, interaction, function, cellular localization and dynamics based on the amino acid sequence [18,47–52]. In this work, the sequence and predicted structure information of BVL-I and -II were used to identify patterns in the C-terminal peptide of single chain lectins.

The SM program excluded some amino acids from the analysed lectins. This exclusion is part of the SM algorithm, which uses the carbon backbone of a template structure with high identity to determine which amino acids in the query sequence are included in the structural analysis. This explains the good RMSD and quality measurements observed, in agreement with previously published data [53–55]. Of note, the excluded amino acids were mainly localised in the C-terminal region. Therefore, this was considered a negative factor for structural studies of this kind of lectin.

An advantage of using the 3DJ algorithm is that it should maintain more amino acids in the query sequence than the SM. However, the overall predicted structure was not accurate since the characteristic legume lectin β-sheets were not predicted for BVL-I. The conserved β-sheets were only included in the predicted 3D model for the SBA lectin. In addition, 3DJ could not predict the structures of BVL-II, EcorL, PNA and DBL, since most of their amino acids were excluded. This is in contrast to reports in the literature where the 3DJ alignment algorithm generated the best results for homology modelling of membrane proteins [56].

Unlike SM and 3DJ, the BH software did not exclude any amino acid residues. The software has a hybrid algorithm with both a homology-based and *ab initio* prediction. Thus, the predicted structures contained the conserved legume lectin β-sheets. However, a C-terminal region of approximately 15 amino acids did not exhibit the type of folding predicted for the rest of the structure. Of the three programs, BH proved to be the most reliable for predicting the structure of single chain lectins. There is little information in the literature on using this program to generate models to study proteins [41]. However, its *ab initio* methodology has been successfully applied to small proteins [57,58].

In the lectins evaluated in this study (SBA, PNA, EcorL and DBL), a C-terminal post-translational modification has been documented, although it is poorly understood. Although no conserved or unique cleavage sites have been described [20], the modification was therefore considered an enzymatic cleavage event [24]. This processing was characterized by the removal of a C-terminal region containing between 12 and 20 amino acid residues [21]. The hydrophobic nature of this peptide is responsible for targeting some lectins to the plant vacuole, suggesting that this region may have the same function in many lectins, including those in this study [19]. Although various approaches have been developed to predict post-translational modifications, they are sequence-based algorithms that detect glycosylation, acetylation, phosphorylation and other kinds of linked molecules [59–63]. This study is the first to report the use of sequence analysis together with structure prediction and reliability evaluation algorithms to detect post-translational cleavage patterns in proteins.

By analysing the C-terminal regions of SBA, EcorL, PNA and DBL it was possible to define three rules for the prediction of the cleavage sites for BVL-I and -II. Namely: (1) the first amino acid of the excised peptide is small or hydrophobic; (2) the cleavage occurs after an acid, polar, or hydrophobic residue, but not after a basic one; and (3) the cleavage spot is located 5-8 residues after a conserved Leu amino acid. Based on sequence alignment, GS-IV appeared to be processed after the basic amino acid Lys. However, cDNA information is not available for this lectin and thus it is not clear whether the reported protein sequence is the truncated form. If this GS-IV protein sequence represents the truncated form, it opens a new possibility for the second rule, permitting a cleavage after a basic amino acid. Comparing GS-IV to the BVL sequences, and considering that they are highly identical, the predicted cleavage spot for BVL-I and -II could also be after the Lys residue. Nevertheless, it would break the third rule, which seems to be unbreakable, as even GS-IV follows it. It is possible that an Asn residue in the region between the conserved Lys and the excised peptide has been deleted in BVL, what would shorten the distance postulated for the third rule to four amino acids. Thus, the deleted Asn in the BVL sequences may play an important role in changing the cleavage spot to approximately two amino acids upstream the site of deletion.

To verify the influence of the C-terminal region on the BVL-I and -II structures, predictions were made with and without the 15 amino acids of this region. The BH structure predictions for BVL-I/BH2 and BVL-II/BH2 included coherent β-sheets, suggesting that the software could reliably predict these structures. Comparing the local errors of the structures with and without the C-terminal region of BVL-I, it was found that the truncated protein sequence contained fewer local errors. This was also noted in the predicted structures for SBA, EcorL and PNA, all of which are known to undergo cleavage in this region [20,24]. However, the local error in the C-terminal region of BVL-II/BH2 was higher than that for BVL-II/BH1, indicating that this portion could potentially have a different function to that of BVL-I.

Although the C-terminal region of BVL-II/BH1 was predicted to be a loop structure, one of the four alternative structures (BVL-II/BHα) adopted a different conformation. Possibly due to the presence of Leu and absence of Ile and Asn in this region of the BVL-II, SBA and DBL C-terminal sequences, which contributed to the formation of the α-helix [44]. Interestingly, the other predictions for BVL-I, EcorL and PNA did not show this characteristic, supporting the hypothesis that BVL-I has different properties to that of BVL-II. Additionally, the presence of an α-helix diminished the local error in the C-terminal region of BVL-II. Therefore, the predicted structural patterns of BVL-II were comparable to those of DBL as they both showed the same reduced C-terminal local error in the presence of an α-helix at this region.

DBL has two isoforms with identical amino acid sequences and the difference in the molecular mass is due to the post-translational cleavage of 12 amino acids at the C-terminus [22,23]. Both variants are found in the dimer and tetramer, which includes a C-terminal α-helix from the intact isoform in the centremost regions in order to stabilize the quaternary structure [64]. Similar to DBL, the SBA lectin forms a tetramer with truncated and non-truncated variants and the predicted structure of SBA/BH included a C-terminal α-helix, correlating with the C-terminal amino acids that were implied by X-ray diffraction [65]. Due to the high local error in the C-terminal region of BVL-I, it appeared to be cleaved, while BVL-II appeared to be intact due to the low local error of the C-terminal region in the presence of an α-helix. This suggested that they may have the same quaternary association characteristics of DBL and SBA. However, both the DBL and SBA tetramers are formed by the interaction of two conserved motifs [18], which are not present in either BVL-I or -II. Instead, BVLs sequences included four conserved regions that corresponded to a GS-I-like (X4+unusual) quaternary association (Figure 1). This data is in accordance with the previous prediction of this same kind of association for the *B. purpurea* lectin (BPA), which has a high sequence identity to BVL-I and -II [18]. As GS-I forms tetramers based on any combination of its two isoforms [66], the BVLs are likely to behave in a similar way. In this case, the tetramers might be formed by any combination of BVL-I and -II. Curiously, the similarity and identity levels between the two GS-I isoforms are similar to those of BVL-I and -II (Table S2). By analysing the structural properties of the BVLs, we observed that a different type of quaternary structure could be formed since (1) both lectins displayed a GS-I-like pattern of quaternary association, and (2) the C-terminal region of BVL-II seemed to assume an α-helical structure which was similar to the DBL and SBA intact isoforms. This unusual evidence of quaternary association was supported by the absence of a C-terminal α-helix among the five possible structures predicted by BH for the BVL-I, EcorL and PNA lectins. Moreover, the role of the high Ser content (five residues) in the C-terminal peptide of both BVL-I and -II should be investigated.

The amino acid sequence data obtained by Edman degradation confirmed that BVL-I could assume a truncated form. The C-terminal 15 amino acids were not present in the mature lectin, in agreement with the predicted cleavage site being between Ser248 and Ala249. By an initial comparison of the intact structures of BVLs, it seems that cleavage may also occur with BVL-II, as it showed similar patterns of local error in this region. However, the presence of an α-helix in the C-terminal region could stabilize this structure and thus characterize BVL-II as an intact form. Immediately upstream the conserved Leu242, BVL-I contained a glycosylation site that is not present in any other lectin but BPA. Glycosylation has been described as a mechanism to avoid protein cleavage in some proteins [67]; however, in BVL-I, the location of the carbohydrate did not seem to be directly involved with the cleavage site. Instead, adding carbohydrates at this specific location of BVL-I may alter the properties of the C-terminal region and serve as marker for the catalytic enzyme in a mechanism similar to that reported for ConA, which is cleaved near to an N-glycosylation site [7,8]. Modifications of the sequence and structure due to C-terminal cleavage can alter lectin function, which is related to the carbohydrate specificity [6,64,68]. Further studies on this kind of processing are needed to fully understand the biological functions of the single chain lectins such as BVL-I and -II.

Although single chain legume lectins are extensively described in literature, the mechanism of their post-translational cleavage is poorly understood. The results presented here suggest that the cleavage site of the C-terminal peptide depends on the properties and location of the amino acids, and not on a conserved region. The three rules proposed in this study may assist analyses of other lectins from this group. To date, the structures of the BVL-I and -II lectins have not been resolved experimentally as these proteins are difficult to crystallise due to the high level of glycosylation and the presence of different isoforms in quaternary association. The *in silico* analysis presented in this study supports that the quaternary associations of the BVLs appeared to be a factor in resolving their structures. This data is in agreement with the fact that the published structures of GS-I only show the quaternary structures with identical subunits (GS-IA or GS-IB) [69]. Indeed, the only member of the Caesalpinioideae subfamily to have a successfully crystallised lectin is *G. simplicifolia*, suggesting this procedure is complicated for related lectins such as the BVLs and BPA. Assuming that the quaternary association of BVL-I and -II is similar to that of GS-I, the purification, crystallization and associated procedures used to resolve the structure of this lectin may be applicable to the BVLs.

## Supporting Information

Figure S1 **Comparison of the characteristic legume lectin β-sheets between the GS-IV and BVL predictions.** The BVL-I and BVL-II amino acid sequences were used as the query sequences in the SM, 3DJ and BH programs. The resulting structures were analysed for the presence of the expected β-sheets: front sheet (red), back sheet (yellow), and small sheet (blue). Since GS-IV had a high sequence identity with both BVL-I and -II, its PDB structure was used as the reference structure. With the exception of the 3DJ predictions, all the other structures contained coherent β-sheets. The 3DJ predictions for BVL-II lacked many amino acids and were not included. (A) GS-IV (PDB entry: 1LEC). (B) BVL-I/SM. (C) BVL-I/3DJ1. (D) BVL-I/3DJ2 (E) BVL-I/BH1. (F) BVL-I/BH2. (G) BVL-II/SM. (H) BVL-II/BH1. (I) BVL-II/BH2. (J) BVL-II/BHα with a fault on the front sheet and the C-terminal α-helix (magenta).
(TIF)

Figure S2 **Comparison of the β-sheets between the PDB structures and their predictions containing the C-terminal peptide.** All of the analysed PDB structures are known to be cleaved at the C-terminal region. Therefore, predicted structures were generated to contain these regions. The resulting models were compared to their corresponding PDB structures to analyse the β-sheets coherence: front sheet (red), back sheet (yellow), and small sheet (blue). The 3DJ predictions for the EcorL, PNA and DBL lectins lacked many amino acids and were not included. All the remainder structures contained coherent β-sheet predictions. (A) SBA (PDB entry: 1SBF). (B) SBA/SM. (C) SBA/3DJ. (D) SBA/BH. (E) EcorL (PDB entry: 1AX0). (F) EcorL/SM. (G) EcorL/BH. (H) PNA (PDB entry: 1CIW). (I) PNA/SM. (J) PNA/BH. (K) DBL:A (PDB entry: 1BJQ, chain A). (L) DBL:C (PDB entry: 1BJQ, chain C). (M) DBL/SM. (N) DBL/BH. Magenta, C-terminal α-helix.
(TIF)

Table S1 **Entry codes for amino acid sequence and protein structure acquisition of the analysed lectins.** All protein sequences and structures were acquired from GenBank and Protein Data Bank (PDB), respectively. Modifications of the protein sequences used as the query sequences are described in the main article.
(DOCX)

Table S2 **Similarity and identity between the analysed lectins sequences.** Sequences were acquired from GenBank and analysed by EMBOSS Needle using the BLOSUM62 matrix [36]. .
(DOCX)

Table S3 **Reliability values for predictions and PDB structures.** The use of four reliability parameters (Z-score, QMEAN score, RP and RMSD) identified the best of the five predictions for each lectin made by the BH program.
(DOCX)

Table S4 **Calculation of the average reliability values of each program for the selection of the best one.** The average values of four reliability parameters (Z-score, QMEAN score, RP and RMSD) were used to select the best prediction program.
(DOCX)

Table S5 **Characteristics of the cleaved C-terminal peptide in the analysed lectins.** The amino acid content of the cleaved C-terminal peptide tends to divide these lectins into two distinct groups: one composed by EcorL and PNA; and other by DBL and SBA. BVL-I and -II are most likely to be part of the second group.
(DOCX)

Text S1 **Protein sequence alignment of the studied lectins with amino acid classification.** The studied lectins were aligned by Clustal Omega, which classified the amino acids into four groups: hydrophobic and small (red), acid (blue), basic (magenta), and polar (green). This classification facilitated the prediction of the cleavage sites.
(DOCX)
