# Supplementary figures and images for: Structure Predictions of Two Bauhinia variegata Lectins Reveal Patterns of C-Terminal Properties in Single Chain Legume Lectins
Source: PLoS One. 2013 Nov 19;8(11):e81338. doi: 10.1371/journal.pone.0081338 (PMC3834338; doi:10.1371/journal.pone.0081338)

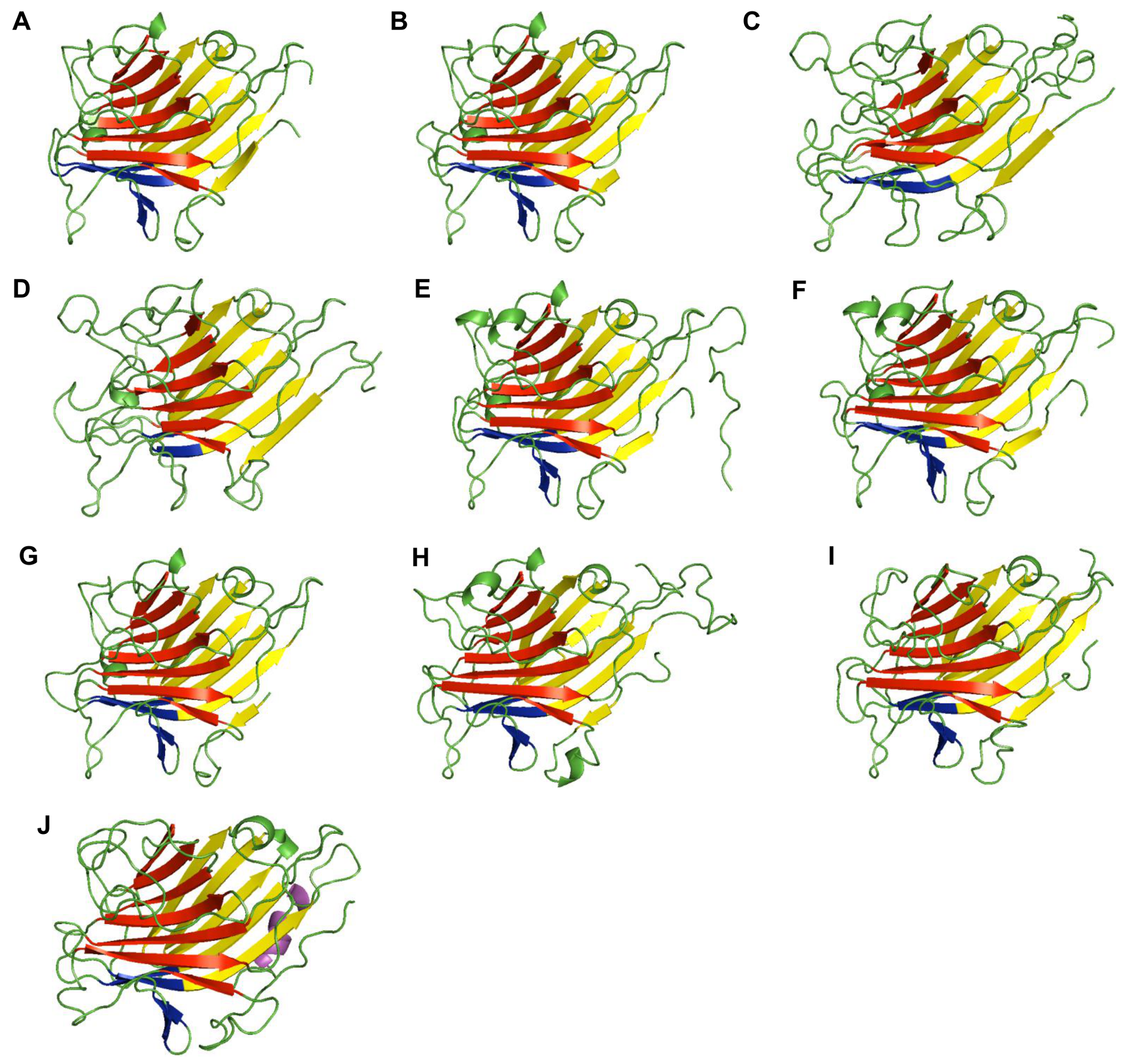

Supplement: Figure S1 — Comparison of the characteristic legume lectin β-sheets between the GS-IV and BVL predictions. The BVL-I and BVL-II amino acid sequences were used as the query sequences in the SM, 3DJ and BH programs. The resulting structures were analysed for the presence of the expected β-sheets: front sheet (red), back sheet (yellow), and small sheet (blue). Since GS-IV had a high sequence identity with both BVL-I and -II, its PDB structure was used as the reference structure. With the exception of the 3DJ predictions, all the other structures contained coherent β-sheets. The 3DJ predictions for BVL-II lacked many amino acids and were not included. (A) GS-IV (PDB entry: 1LEC). (B) BVL-I/SM. (C) BVL-I/3DJ1. (D) BVL-I/3DJ2 (E) BVL-I/BH1. (F) BVL-I/BH2. (G) BVL-II/SM. (H) BVL-II/BH1. (I) BVL-II/BH2. (J) BVL-II/BHα with a fault on the front sheet and the C-terminal α-helix (magenta). (TIF) [file pone.0081338.s001.tif]

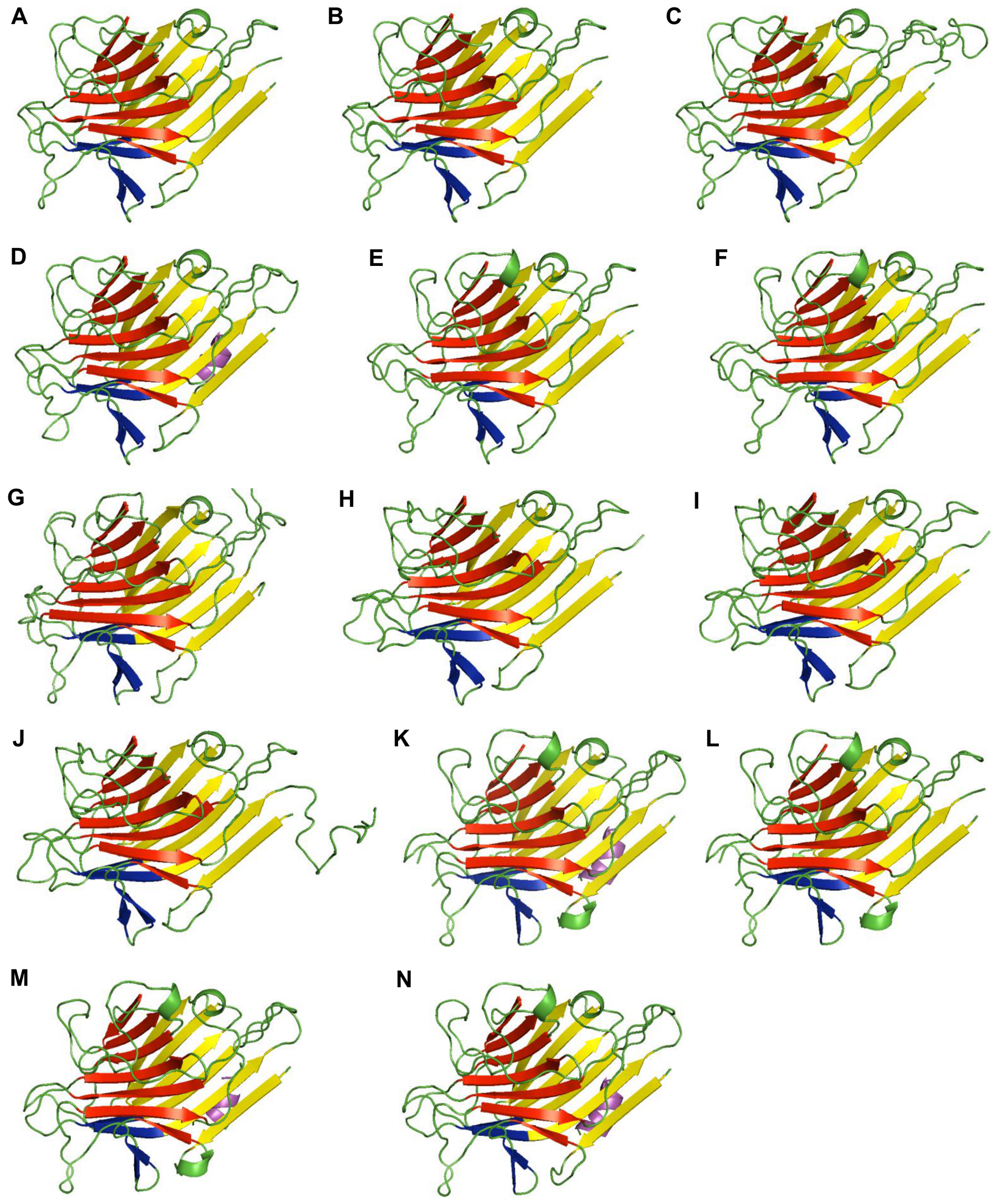

Supplement: Figure S2 — Comparison of the β-sheets between the PDB structures and their predictions containing the C-terminal peptide. All of the analysed PDB structures are known to be cleaved at the C-terminal region. Therefore, predicted structures were generated to contain these regions. The resulting models were compared to their corresponding PDB structures to analyse the β-sheets coherence: front sheet (red), back sheet (yellow), and small sheet (blue). The 3DJ predictions for the EcorL, PNA and DBL lectins lacked many amino acids and were not included. All the remainder structures contained coherent β-sheet predictions. (A) SBA (PDB entry: 1SBF). (B) SBA/SM. (C) SBA/3DJ. (D) SBA/BH. (E) EcorL (PDB entry: 1AX0). (F) EcorL/SM. (G) EcorL/BH. (H) PNA (PDB entry: 1CIW). (I) PNA/SM. (J) PNA/BH. (K) DBL:A (PDB entry: 1BJQ, chain A). (L) DBL:C (PDB entry: 1BJQ, chain C). (M) DBL/SM. (N) DBL/BH. Magenta, C-terminal α-helix. (TIF) [file pone.0081338.s002.tif]
