# Supplementary material for: Structure Predictions of Two Bauhinia variegata Lectins Reveal Patterns of C-Terminal Properties in Single Chain Legume Lectins
Source: PLoS One. 2013 Nov 19;8(11):e81338. doi: 10.1371/journal.pone.0081338 (PMC3834338; doi:10.1371/journal.pone.0081338)
Supplement: Table S1 — Entry codes for amino acid sequence and protein structure acquisition of the analysed lectins. All protein sequences and structures were acquired from GenBank and Protein Data Bank (PDB), respectively. Modifications of the protein sequences used as the query sequences are described in the main article. (DOCX) [file pone.0081338.s003.docx]

Table S1 Entry codes for amino acid sequence and protein structure acquisition of the analysed lectins.

| Protein ID | Accession number  (GenBank) | Structure  (PDB) |
| --- | --- | --- |
| BVL-I | ABQ45362.1 | N.R. |
| BVL-II | ACB87491.1 | N.R. |
| GS-IV | P24146.3 | 1LEC |
| SBA | P05046.1 | 1SBF |
| EcorL | CAA36986.1 | 1AX0 |
| PNA | AAB22817.1 | 1CIW |
| DBL | P05045.2 | 1BJQ^a^ |
| BPA | BAA02049.1 | N.R. |
| GS-IA | AAL65146.1 | N.A. |
| GS-IB | AAL65147.1 | N.A. |

N.R., not resolved.

N.A., not analysed.

^a^Two structures were acquired from 1BJQ, one is DBL with the C-terminal region (chain A, called DBL:A), and the other is DBL without the C-terminal region (chain C, called DBL:C).
