# Supplementary material for: Structure Predictions of Two Bauhinia variegata Lectins Reveal Patterns of C-Terminal Properties in Single Chain Legume Lectins
Source: PLoS One. 2013 Nov 19;8(11):e81338. doi: 10.1371/journal.pone.0081338 (PMC3834338; doi:10.1371/journal.pone.0081338)
Supplement: Table S2 — Similarity and identity between the analysed lectins sequences. Sequences were acquired from GenBank and analysed by EMBOSS Needle using the BLOSUM62 matrix [36]. . (DOCX) [file pone.0081338.s004.docx]

Table S2 Similarity and identity between the analysed lectins sequences.

| % Identity | % Similarity | | | | | | | | | |
| --- | --- | --- | --- | --- | --- | --- | --- | --- | --- | --- |
|  | BVL-I^a^ | BVL-II^a^ | GS-IV | SBA | EcorL | PNA | DBL | BPA | GS-IA | GS-IB |
| BVL-I | **100** | 92.8 | 75.7 | 54.2 | 53.3 | 52.0 | 51.4 | 98.1 | 64.7 | 61.5 |
| BVL-II | 87.8 | **100** | 74.1 | 55.4 | 52.6 | 52.4 | 51.7 | 91.6 | 64.7 | 62.9 |
| GS-IV | 63.1 | 64.6 | **100** | 52.0 | 51.5 | 51.7 | 51.9 | 75.7 | 63.3 | 62.9 |
| SBA | 41.0 | 38.1 | 41.3 | **100** | 65.3 | 53.7 | 71.3 | 54.8 | 54.2 | 55.1 |
| EcorL | 34.7 | 34.6 | 36.1 | 48.6 | **100** | 56.0 | 60.4 | 53.8 | 51.5 | 53.9 |
| PNA | 35.5 | 35.4 | 35.2 | 38.5 | 39.1 | **100** | 54.4 | 53.3 | 46.7 | 45.8 |
| DBL | 38.8 | 38.5 | 41.1 | 57.8 | 42.7 | 34.4 | **100** | 51.8 | 55.4 | 56.1 |
| BPA | 98.1 | 86.7 | 62.7 | 41.5 | 35.5 | 37.1 | 38.8 | **100** | 65.1 | 61.8 |
| GS-IA | 50.9 | 51.1 | 54.4 | 41.9 | 38.6 | 31.7 | 38.3 | 50.9 | **100** | 92.8 |
| GS-IB | 48.0 | 48.4 | 52.1 | 41.7 | 38.7 | 30.9 | 39.4 | 48.0 | 88.4 | **100** |

^a^Sequences acquired from GenBank, without the 28 amino acid from the N-terminal signal peptide.
