# Supplementary material for: Structure Predictions of Two Bauhinia variegata Lectins Reveal Patterns of C-Terminal Properties in Single Chain Legume Lectins
Source: PLoS One. 2013 Nov 19;8(11):e81338. doi: 10.1371/journal.pone.0081338 (PMC3834338; doi:10.1371/journal.pone.0081338)
Supplement: Table S3 — Reliability values for predictions and PDB structures. The use of four reliability parameters (Z-score, QMEAN score, RP and RMSD) identified the best of the five predictions for each lectin made by the BH program. (DOCX) [file pone.0081338.s005.docx]

Table S3 Reliability values for predictions and PDB structures.

| Structure | Z-score^a^ | QMEAN score^b^ | RP (%)^c^ | RMSD (Å)^d^ |
| --- | --- | --- | --- | --- |
| BVL-I/SM | -0.90 | 0.73 | 86.9 | 0.41^e^ |
| BVL-I/3DJ1 | -1.89 | 0.59 | 63.9 | 2.73 |
| BVL-I/3DJ2 | -1.67 | 0.61 | 70.2 | 2.79 |
| BVL-I/BH | -1.44 | 0.63 | 78.3 | 3.23 |
| BVL-I/BH | -1.45 | 0.63 | 79.6 | 3.10 |
| BVL-I/BH | -6.09 | 0.20 | 63.9 | 18.28 |
| BVL-I/BH | -6.23 | 0.19 | 67.8 | 17.92 |
| **BVL-I/BH1** | **-1.36** | **0.64** | **90.9** | **0.25** |
| BVL-I/BH2 | -2.38 | 0.55 | 75.3 | 6.92 |
| BVL-I/BH2 | -0.81 | 0.69 | 78.6 | 3.14 |
| BVL-I/BH2 | -1.24 | 0.65 | 85.6 | 0.46 |
| BVL-I/BH2 | -1.52 | 0.63 | 87.0 | 2.55 |
| **BVL-I/BH2** | **-1.05** | **0.67** | **85.6** | **0.43** |
| BVL-II/SM | -0.54 | 0.75 | 83.7 | 0.69^e^ |
| BVL-II/3DJ1 | -3.30 | 0.44 | 93.8 | 7.61 |
| BVL-II/3DJ2 | -2.84 | 0.47 | 90.9 | 7.64 |
| BVL-II/BH1 | -2.30 | 0.55 | 73.3 | 7.08 |
| BVL-II/BH1 | -2.80 | 0.51 | 75.9 | 10.88 |
| BVL-II/BH1 | -3.12 | 0.48 | 75.9 | 8.55 |
| BVL-II/BHα | -2.32 | 0.55 | 78.0 | 2.31 |
| **BVL-II/BH1** | **-1.28** | **0.65** | **86.2** | **2.11** |
| BVL-II/BH2 | -2.76 | 0.51 | 73.7 | 5.62 |
| BVL-II/BH2 | -1.59 | 0.62 | 79.3 | 2.39 |
| BVL-II/BH2 | -1.37 | 0.64 | 83.4 | 2.56 |
| BVL-II/BH2 | -1.86 | 0.60 | 77.9 | 3.18 |
| **BVL-II/BH2** | **-0.93** | **0.68** | **86.6** | **0.54** |
| GS-IV | 1.43 | 0.90 | 88.6 | **---** |
| SBA/SM | 0.01 | 0.77 | 84.8 | 0.09^e^ |
| SBA/3DJ | -0.66 | 0.71 | 75.2 | 0.23 |
| SBA/BH | -2.02 | 0.58 | 73.9 | 8.47 |
| SBA/BH | -0.30 | 0.74 | 79.3 | 1.87 |
| SBA/BH | -7.47 | 0.07 | 64.0 | 19.66 |
| SBA/BH | -1.71 | 0.61 | 82.9 | 6.52 |
| **SBA/BH** | **-0.65** | **0.71** | **90.5** | **0.59** |
| SBA | 0.47 | 0.81 | 86.3 | **---** |
| EcorL/SM | 0.15 | 0.88 | 86.2 | 0.06^e^ |
| EcorL/3DJ | -1.03 | 0.66 | 90.0 | 0.93 |
| EcorL/BH | -1.06 | 0.67 | 81.7 | 2.49 |
| EcorL/BH | -1.30 | 0.65 | 76.1 | 2.56 |
| EcorL/BH | -0.83 | 0.69 | 78.0 | 1.34 |
| EcorL/BH | -1.54 | 0.62 | 78.4 | 2.88 |
| **EcorL/BH** | **0.37** | **0.80** | **88.1** | **1.67** |
| EcorL | 1.69 | 0.93 | 90.1 | **---** |
| PNA/SM | 0.72 | 0.81 | 90.5 | 0.19^e^ |
| PNA/3DJ | -0.43 | 0.71 | 90.7 | 5.02 |
| PNA/BH | -2.15 | 0.57 | 78.0 | 5.99 |
| PNA/BH | -3.16 | 0.48 | 78.0 | 7.89 |
| PNA/BH | -6.07 | 0.20 | 65.6 | 18.92 |
| PNA/BH | -7.05 | 0.11 | 71.6 | 19.36 |
| **PNA/BH** | **0.51** | **0.82** | **91.7** | **0.42** |
| PNA | 1.64 | 0.92 | 92.0 | **---** |
| DBL/SM | 0.59 | 0.73 | 82.5 | 0.11^e^ |
| DBL/3DJ | -2.83 | 0.49 | 91.7 | 2.70 |
| DBL/BH | -3.36 | 0.45 | 72.2 | 10.82 |
| DBL/BH | -1.65 | 0.62 | 92.4 | 6.89 |
| DBL/BH | -6.26 | 0.18 | 66.8 | 18.68 |
| DBL/BH | -0.65 | 0.71 | 88.8 | 0.37 |
| **DBL/BH** | **-0.42** | **0.73** | **91.9** | **0.31** |
| DBL:A | -0.38 | 0.73 | 89.5 | **---** |
| DBL:C | -0.20 | 0.75 | 90.1 | **---** |

Bold, selected structures from Bhageerath-H.

^a^ Expected Z-score is: |Z-score|<1 for good predictions, 1<|Z-score|<2 for medium predictions, and |Z-score|>2 for bad predictions.

^b^ Expected QMEAN score is ≈1.

^c^ Expected RP value is >90%, but the values form PDB structures were used as reference.

^d^ Expected RMSD value is <2.5Å.

^e^ Expected RMSD value is <1Å.
