# Supplementary material for: Structure Predictions of Two Bauhinia variegata Lectins Reveal Patterns of C-Terminal Properties in Single Chain Legume Lectins
Source: PLoS One. 2013 Nov 19;8(11):e81338. doi: 10.1371/journal.pone.0081338 (PMC3834338; doi:10.1371/journal.pone.0081338)
Supplement: Table S4 — Calculation of the average reliability values of each program for the selection of the best one. The average values of four reliability parameters (Z-score, QMEAN score, RP and RMSD) were used to select the best prediction program. (DOCX) [file pone.0081338.s006.docx]

Table S4 Calculation of the average values of reliability for the selection of the best prediction program.

| Program | Structure ID | Reliability Values | | | |
| --- | --- | --- | --- | --- | --- |
| SwissModel |  | Z-score^a^ | QMEAN score^b^ | RP (%)^c^ | RMSD (Å)^d^ |
|  | BVL-I/SM | -0.90 | 0.73 | 86.9 | 0.41^e^ |
|  | BVL-II/SM | -0.54 | 0.75 | 83.7 | 0.69^e^ |
|  | SBA/SM | 0.01 | 0.77 | 84.8 | 0.09^e^ |
|  | EcorL/SM | 0.15 | 0.88 | 86.2 | 0.06^e^ |
|  | PNA/SM | 0.72 | 0.81 | 90.5 | 0.19^e^ |
|  | DBL/SM | 0.59 | 0.73 | 82.5 | 0.11^e^ |
|  | Avg.^f^ | 0.49 | 0.78 | 85.8 | 0.26^e^ |
| 3D Jigsaw |  |  |  |  |  |
|  | BVL-I/3DJ1 | -1.89 | 0.59 | 63.9 | 2.73 |
|  | BVL-I/3DJ2 | -1.67 | 0.61 | 70.2 | 2.79 |
|  | SBA/3DJ | -0.66 | 0.71 | 75.2 | 0.23 |
|  | Avg.^f^ | 1.41 | 0.64 | 69.8 | 1.92 |
| Bhageerath-H |  |  |  |  |  |
|  | BVL-I/BH1 | -1.36 | 0.64 | 90.9 | 0.25 |
|  | BVL-I/BH2 | -1.05 | 0.67 | 85.6 | 0.43 |
|  | BVL-II/BH1 | -1.28 | 0.65 | 86.2 | 2.11 |
|  | BVL-II/BH2 | -0.93 | 0.68 | 86.6 | 0.54 |
|  | SBA/BH | -0.65 | 0.71 | 90.5 | 0.59 |
|  | EcorL/BH | 0.37 | 0.80 | 88.1 | 1.67 |
|  | PNA/BH | 0.51 | 0.82 | 91.7 | 0.42 |
|  | DBL/BH | -0.42 | 0.73 | 91.9 | 0.31 |
|  | Avg.^f^ | 0.82 | 0.71 | 88.9 | 0.79 |
| PDB |  |  |  |  |  |
|  | GS-IV | 1.43 | 0.90 | 88.6 | **---** |
|  | SBA | 0.47 | 0.81 | 86.3 | **---** |
|  | EcorL | 1.69 | 0.93 | 90.1 | **---** |
|  | PNA | 1.64 | 0.92 | 92.0 | **---** |
|  | DBL:A | -0.38 | 0.73 | 89.5 | **---** |
|  | DBL:C | -0.20 | 0.75 | 90.1 | **---** |
|  | Avg.^f^ | 0.88 | 0.83 | 89.6 | **---** |

^a^ Expected Z-score is: |Z-score|<1 for good predictions, 1<|Z-score|<2 for medium predictions, and |Z-score|>2 for bad predictions.

^b^ Expected QMEAN score is ≈1.

^c^ Expected RP value is >90%, but the values form PDB structures were used as reference.

^d^ Expected RMSD value is <2.5Å.

^e^ Expected RMSD value is <1Å.

^f^ Average, which was calculated as the arithmetic mean of each value. For Z-score, the module of the values were summed and then divided.
