# Supplementary material for: Structure Predictions of Two Bauhinia variegata Lectins Reveal Patterns of C-Terminal Properties in Single Chain Legume Lectins
Source: PLoS One. 2013 Nov 19;8(11):e81338. doi: 10.1371/journal.pone.0081338 (PMC3834338; doi:10.1371/journal.pone.0081338)
Supplement: Table S5 — Characteristics of the cleaved C-terminal peptide in the analysed lectins. The amino acid content of the cleaved C-terminal peptide tends to divide these lectins into two distinct groups: one composed by EcorL and PNA; and other by DBL and SBA. BVL-I and -II are most likely to be part of the second group. (DOCX) [file pone.0081338.s007.docx]

**Table S5 Characteristics of the cleaved C-terminal peptide in the analysed lectins.**

| **Lectin** | **Nº of Asn** | **Nº of Leu** | **Nº of Ile** | **Buried first Leu** |
| --- | --- | --- | --- | --- |
| EcorL | 2 | 0 | 2 | No^a^ |
| PNA | 3 | 0 | 2 | No^a^ |
| DBL | 1 | 3 | 1 | Yes |
| SBA | 0 | 4 | 0 | Yes |
| BVL-I and -II | 0 | 2 | 0 | Yes^b^ |

^a^ The lectins actually do not contain this Leu.

^b^ BVL-I has no C-terminal peptide.
