# Supplementary material for: Structure Predictions of Two Bauhinia variegata Lectins Reveal Patterns of C-Terminal Properties in Single Chain Legume Lectins
Source: PLoS One. 2013 Nov 19;8(11):e81338. doi: 10.1371/journal.pone.0081338 (PMC3834338; doi:10.1371/journal.pone.0081338)
Supplement: Text S1 — Protein sequence alignment of the studied lectins with amino acid classification. The studied lectins were aligned by Clustal Omega, which classified the amino acids into four groups: hydrophobic and small (red), acid (blue), basic (magenta), and polar (green). This classification facilitated the prediction of the cleavage sites. (DOCX) [file pone.0081338.s008.docx]

BVL-I 1 TSSTLTGFTFPNFWSNTQENGTEIIFLGNATY-TPGALRLTRIGEDGIPLKSNAGQASYS 59

BVL-II 1 TSSTLTSFTFPDFWSNSQENGTKIIFLGGATY-TPGALRLTRIAKDGFPMKSNAGQASYS 59

GS-IV 1 --QNTVNFTYPDFWSYSLKNGTEITFLGDATR-IPGALQLTKTDANGNPVRSSAGQASYS 57

SBA 1 --AETVSFSWNKF----VPKQPNMILQGDAIVTSSGKLQLNKVDENGTPKPSSLGRALYS 54

EcorL 1 --VETISFSFSEF----EPGNDNLTLQGAALITQSGVLQLTKINQNGMPAWDSTGRTLYA 54

PNA 1 --AETVSFNFNSF----SEGNPAINFQGDVTVLSNGNIQLTNLNK-----VNSVGRVLYA 49

DBL 1 --ANIQSFSFKNF------NSPSFILQGDATV-SSGKLQLTKVKENGIPTPSSLGRAFYS 51

BPA 1 TSSTLTGFTFPNFWSNTQENGTEIIFLGNATY-TPGALRLTRIGEDGIPLKSNAGQASYS 59

GS-IA 1 -------FNLPNFWSDVK---DNIIFQGDANT-TAGTLQLCKTNQYGNPLQYRAGRALYS 49

GS-IB 1 -QSDSVSFTFPNFWSDVE---DSIIFQGDANT-TAGTLQLCKTNQYGTPLQWSAGRALYS 55

*. .* : : * . * ::* . *:. *:

BVL-I 60 RPVFLWDS-TGHVASFYTSFSFIVRSIDVPHITADGFASFLAPVDSSVKD---YGGCLGL 115

BVL-II 60 HPVFLWDS-TGHVASFYTSFSFIVRNCDVPKITADGFAFFLAPVDSSVKG---FGGCLGL 115

GS-IV 58 EPVFLWDS-TGKAASFYTSFTFLLKNYGAP--TADGLAFFLAPVDSSVKD---YGGFLGL 111

SBA 55 TPIHIWDKETGSVASFAASFNFTFYAPDTKR-LADGLAFFLAPIDTKPQT---HAGYLGL 110

EcorL 55 KPVHIWDMTTGTVASFETRFSFSIEQPYTRPLPADGLVFFMGPTKSKPAQ---GYGYLGI 111

PNA 50 MPVRIWSSATGNVASFLTSFSFEMKDIKDYD-PADGIIFFIAPEDTQIPAGSIGGGTLGV 108

DBL 52 SPIQIYDKSTGAVASWATSFTVKISAPSKAS-FADGIAFALVPVGSEPRR---NGGYLGV 107

BPA 50 RPVFLWDS-TGHVASFYTSFSFIVRSIDVPHITADGFAFFLAPVDSSVKD---YGGCLGL 115

GS-IA 50 DPVQLWDNKTGSVASFYTEFTFFLKITGDG--PADGLAFFLAPPDSDVKD---AGAYLGL 104

GS-IB 56 DPVQLWDNKTESVASFYTEFTFFLKITGNG--PADGLAFFLAPPDSDVKD---AGEYLGL 110

*: ::. * .**: : *.. . ***: : * :. **:

BVL-I 116 FRYKTATDPSKNQVVAVEFDTWPNTEWSDLRYPHIGINVNSTVSVATTRWDNDDAYGNKI 175

BVL-II 116 FTYGTAADPSKNQVVAVEFDTWPNTQWSDLSYRHIGIDVNSIVSVATRRWENDDAYGNKI 175

GS-IV 112 FRHETAADPSKNQVVAVEFDTWINKDWNDPPYPHIGIDVNSIVSVATTRWENDDAYGSSI 171

SBA 111 FNENES----GDQVVAVEFDTFR-NSWDP-PNPHIGINVNSIRSIKTTSWDLA---NNKV 161

EcorL 112 FNNSKQ--DNSYQTLGVEFDTFSN-PWDPPQVPHIGIDVNSIRSIKTQPFQLD---NGQV 165

PNA 109 SDT-----KGAGHFVGVEFDTYSNSEYNDPPTDHVGIDVNSVDSVKTVPWNSV---SGAV 160

DBL 108 FDSDVY--NNSAQTVAVEFDTLSNSGWDP-SMKHIGIDVNSIKSIATVSWDLA---NGEN 161

BPA 116 FRYKTATDPSKNQVVAVEFDTWPNTEWSDLRYPHIGINVNSTVSVATTRWDNDDAYVTK- 174

GS-IA 105 FNKSTATQPSKNQVVAVEFDTWKNTDFPEPSYRHIGINVNSIVSVATKRWEDSDIFSGKI 164

GS-IB 111 FNKSTATQPSKNQVVAVEFDTWTNPNFPEPSYRHIGINVNSIVSVATKRWEDSDIFSGKI 170

: :.***** : *:**:*** *: * ::

BVL-I 176 GTAHITYDATSKIITVLLTYDN-GRHYQLSHVVDLPKILPERVRIGFSGGTGFNE----T 230

BVL-II 176 GTAHITYDATSKIITVLLTYDN-GRHYQLSHVVDLPKILPKWVRIGFSAATGYNE----T 230

GS-IV 172 ATAHITYDARSKILTVLLSYEH-GRDYILSHVVDLAKVLPQKVRIGFSAGVGYDE----V 226

SBA 162 AKVLITYDASTSLLVASLVYPSQRTSNILSDVVDLKTSLPEWVRIGFSAATGLDIP-GES 220

EcorL 166 ANVVIKYDASSKILHAVLVYPSSGAIYTIAEIVDVKQVLPEWVDVGLSGATGAQRDAAET 225

PNA 161 VKVTVIYDSSTKTLSVAVTNDN-GDITTIAQVVDLKAKLPERVKFGFSASGSLGG--RQI 217

DBL 162 AEILITYNAATSLLVASLVHPSRRTSYILSERVDITNELPEYVSVGFSATTGLSEGYIET 221

BPA 164 STAHITYDATSKIITVLLTYDN-GRHYQLSHVVDLPKILPERVRIGFSGGTGFNE----T 229

GS-IA 165 ATARISYDGSAKILTVVLSYPD-GADYILSHSVDLSKNLPNPIRVGISASTGANQF--LT 221

GS-IB 171 ATARISYDGSAEILTVVLSYPD-GSDYILSHSVDMRQNLPESVRVGISASTGNNQF--LT 227

: *:. :. : . : ::. **: **: : .*:*. .

BVL-I 231 QYILSWSFTSTLNSTKISALTQKLRSSASYSSM- 263

BVL-II 231 QYILSWSFTSTLDSTKISALTQKLRSSASYSSM- 263

GS-IV 227 TYILSWHFFSTLDGTNK----------------- 243

SBA 221 HDVLSWSFASNLPHASSNIDPLDLT-SFVLHEAI 253

EcorL 226 HDVYSWSFQASLPETNDAVIPTSNHNTFAI---- 255

PNA 218 HLIRSWSFTSTLITTTRRSIDNNEKKIMNMASA- 250

DBL 222 HDVLSWSFASKLPDDS-TAEPLDLA-SYLVRNVL 253

BPA 219 QYILSWSFTSTLNSTKISALTQKLRSSASYSSM- 262

GS-IA 222 VYVLSWRFSSALQSTSVNAAMGPEIIRTVV---- 251

GS-IB 228 VYILSWRFSSNLQSTSVKAAMEPEITRTVV---- 257

: ** * : * .
